# Association between reduced physical performance measures and short-term consequences after major emergency abdominal surgery: a prospective cohort study

**DOI:** 10.1007/s00068-023-02408-4

**Published:** 2024-01-05

**Authors:** Dunja Kokotovic, Aide Schucany, Liv Soylu, Andreas Q. Fenger, Iben Puggard, Sarah Ekeloef, Ismail Gögenur, Jakob Burcharth

**Affiliations:** 1 Department of Gastrointestinal Surgery, Copenhagen University Hospital–Herlev and Gentofte, Copenhagen, Denmark; 2 Emergency Surgery Research Group (EMERGE) Copenhagen, Borgmester Ib Juuls Vej 1, 2730 Herlev, Denmark; 3 Department of Gastrointestinal Surgery, North Zealand University Hospital, Hillerød, Denmark; 4 Department of Physiotherapy, Zealand University Hospital, Køge, Denmark; 5 Center for Surgical Science, Zealand University Hospital, Køge, Denmark

**Keywords:** Emergency surgery, Laparotomy, Postoperative physiotherapy, Postoperative complications, Rehabilitation, DEMMI, Hand grip strength, 30-s chair-stand test

## Abstract

**Background:**

Major emergency abdominal surgery is associated with high morbidity with outcomes worse than for similar elective surgery, including complicated physical recovery, increased need for rehabilitation, and prolonged hospitalisation.

**Purpose:**

To investigate whether low physical performance test scores were associated with an increased risk of postoperative complications, and, furthermore, to investigate the feasibility of postoperative performance tests in patients undergoing major emergency abdominal surgery. We hypothesize that patients with low performance test scores suffer more postoperative complications.

**Methods:**

The study is a prospective observational cohort study including all patients who underwent major abdominal surgery at the Department of Surgery at Zealand University Hospital between 1st March 2017 and 31st January 2019. Patients were evaluated with De Morton Mobility Index (DEMMI) score, hand grip strength, and 30-s chair-stand test.

**Results:**

The study included 488 patients (median age 69, 50.6% male). Physiotherapeutic evaluation including physical performance tests with DEMMI and hand grip strength in the immediate postoperative period were feasible in up to 68% of patients undergoing major emergency abdominal surgery. The 30-s chair-stand test was less viable in this population; only 21% of the patients could complete the 30-s chair-stand test during the postoperative period. In logistic regression models low DEMMI score (< 40) and ASA classification and low hand grip strength (< 20 kg for women, < 30 kg for men were independent risk factors for the development of postoperative severe complications Clavien–Dindo (CD) grade ≥ 3.

**Conclusions:**

In patients undergoing major emergency surgery low performance test scores (DEMMI and hand grip strength), were independently associated with the development of significant postoperative complications CD ≥ 3.

**Supplementary Information:**

The online version contains supplementary material available at 10.1007/s00068-023-02408-4.

## Introduction

Major emergency abdominal surgery is associated with high morbidity and mortality, and the risk of surgical complications is increased compared with similar elective procedures. Patients undergoing emergency laparotomy are in a state of physiological derangement driven by inflammation already occurring prior to surgery, as opposed to patients undergoing elective abdominal procedures. [1]. The postoperative course for patients undergoing major emergency surgery includes prolonged paralytic ileus, complicated physical recovery, increased need for rehabilitation, and prolonged hospitalization [2–5]. Early mobilization and exercise play essential roles in postoperative care following abdominal surgery and are associated with less postoperative reduction of fitness and fewer postoperative complications in patients undergoing elective surgery [6, 7].

Various performance tests can evaluate mobility and indicate sarcopenia and physical frailty. [8] De Morton Mobility Index (DEMMI) score, hand grip strength, and 30-s chair-stand test have been proven helpful in evaluating older adults admitted to the emergency department and associated with length of stay [9–11]. Furthermore, the DEMMI score is associated with the length of stay after general surgery [12]. The performance tests are non-invasive, simple, and quick to perform. However, the feasibility and association of performance tests and postoperative complications have never been investigated in a patient population undergoing major emergency abdominal surgery. Postoperative measures to predict patients at risk for complications and a prolonged postoperative course could support clinical decision-making and assist clinicians in identifying patients in need of increased postoperative initiatives and optimization to improve outcomes.

This study aimed to investigate whether low physical performance test scores were associated with an increased risk of postoperative complications, and furthermore investigate the feasibility of postoperative performance tests in patients undergoing major emergency abdominal surgery. We hypothesize that patients with low performance test scores suffer more postoperative complications compared with patients with high performance scores as a low performance test scores can indicate a reduced physical reserve.

## Methods

### Data source and study population

All patients who underwent major abdominal surgery (emergency laparotomy or laparoscopy (Supplementary Table 1)) at the Department of Surgery at Zealand University Hospital between 1st March 2017 and 31st January 2019 were included. Both patients undergoing laparotomy and laparoscopy were included as this study aimed to investigate the entire group of patients undergoing surgery for bowel obstruction, perforation and ischemia with a focus on the emergency pathophysiology and not specific procedures. The Department of Surgery at Zealand University Hospital is a public referral hospital with a regional specialized role in emergency surgery, bariatric surgery, and colorectal cancer surgery. All patients received a standardized pre-, intra-, and postoperative bundle of care to shorten the time to surgery, remove logistical impediments, and implement best-practice care. The preoperative bundle includes rapid assessment for physiological derangement, early imaging with CT scan with IV contrast, early contact to associates from other departments to initiate and facilitate next steps towards surgery (anaesthesiologist, senior surgeon, operating room and ward personnel), and surgery as soon as possible (within 6 h for patient with sepsis and 12 h for patients without sepsis). The intraoperative bundle included goal directed hemodynamic therapy and a standardized lung ventilation strategy. The standardized postoperative bundle included multimodal systemic analgetics, physiotherapeutic assessment, and standardized nutritional interventions. The physiotherapeutic evaluation was performed at the surgical ward as soon as possible after surgery. For patients admitted to the intensive care unit directly after surgery, the evaluation was performed when the patient returned to the surgical ward. All data were gathered prospectively.

### Physical performance tests

During the standardized physiotherapeutic assessment, performance tests were performed whenever possible and preferably within the first 48 h after return to the department of surgery. The goal was that all patients completed a performance test at the postoperative initiation of physiotherapy. If possible performance test could be repeated at discharge. The performance tests consisted of the De Morton Mobility Index (DEMMI), hand grip strength measurements, and a 30-s chair-stand test.

The DEMMI score is a mobility score and consists of 15 mobility items. Item 13 (bending to pick up a pencil from the floor) and Item 15 (jumping) were not performed because these items were not safe in a newly operated population [13]. A low DEMMI score was defined as DEMMI < 40 [9]. Hand grip strength was measured by a hydraulic hand dynamometer and performed for right and left hands. Grip strength was defined as the maximally measured grip strength of the dominant hand.[14] Low hand grip strength was defined as < 20 kg (kg) for women and < 30 kg for men as proposed by the European Working Group on Sarcopenia in Older People [15]. The 30-s chair-stand test (30 s-CST) is a single-item physical performance tool for assessing lower body strength. It is performed by counting the number of stands completed in 30 s with hands crossed against the chest [16]. Cutoff for a low score on 30 s-CST was defined as < 8 [10]. These tests were chosen to examine a variety of physical functions such as muscle strength in upper and lower body and functional strength and mobility.

### Outcomes

The primary outcome was the association between low postoperative performance and the development of a severe postoperative complication during admission. In-hospital postoperative complications were classified according to the Clavien–Dindo (CD) classification of complications [17], and for the analyses, complications CD grade ≥ 3 were included. CD grade ≥ 3 includes all complications requiring surgical intervention. To secure a temporal association between performance tests and in-hospital complications, only complications registered on a later date than the performance tests were included. This secured at least 24 h between the performance test and the complication. The secondary outcomes were the association of performance tests and the length of stay, the risk of needing postoperative rehabilitation after discharge, and 30-day mortality. The tertiary outcome was the feasibility of performing performance tests early after major emergency abdominal surgery.

The study was approved by the Danish Data Protection Agency (no: REG-042–2017). The study did not qualify for ethics approval by Danish law. The study was conducted and reported in accordance with the Strengthening The Reporting of Observational Studies in Epidemiology (STROBE) checklist [18].

### Statistics

The distribution of continuous data was assessed by visual inspection of histograms. Categorical data were presented as frequencies and percentages. For the continuous variables, median and interquartile range (IQR) were calculated, and the Mann–Whitney *U* test was used for comparison for data not normally distributed, and mean and standard deviation (SD) were calculated for normally distributed data. Differences in categorical variables (binary and polytomous variables) were analyzed with Pearson’s Chi-squared test and Fischer's exact *t* test when appropriate.

To assess whether DEMMI and hand grip strength were independent risk factors for postoperative complications, logistic regression was performed for postoperative complications CD grade ≥ 3. The analyses were adjusted for clinically relevant variables (gender, age (< 60, 60–69, 70–79 or ≥ 80 years of age), performance status (0–1 or ≥ 2), ASA-score (1–2, 3–5), and open or laparoscopic surgery). To include variation regarding the surgical approach, the variable open vs. laparoscopic surgery was chosen as open surgery is suspected of impeding mobilization more than laparoscopic surgery. The ASA score was used as the proxy for patient-related comorbidities. The model was created with one variable per 10 events (complications) to prevent overfitting.

IBM SPSS Statistic 28 for MAC was used to compute the statistics.

## Results

### Demographics

**Fig. 1:**
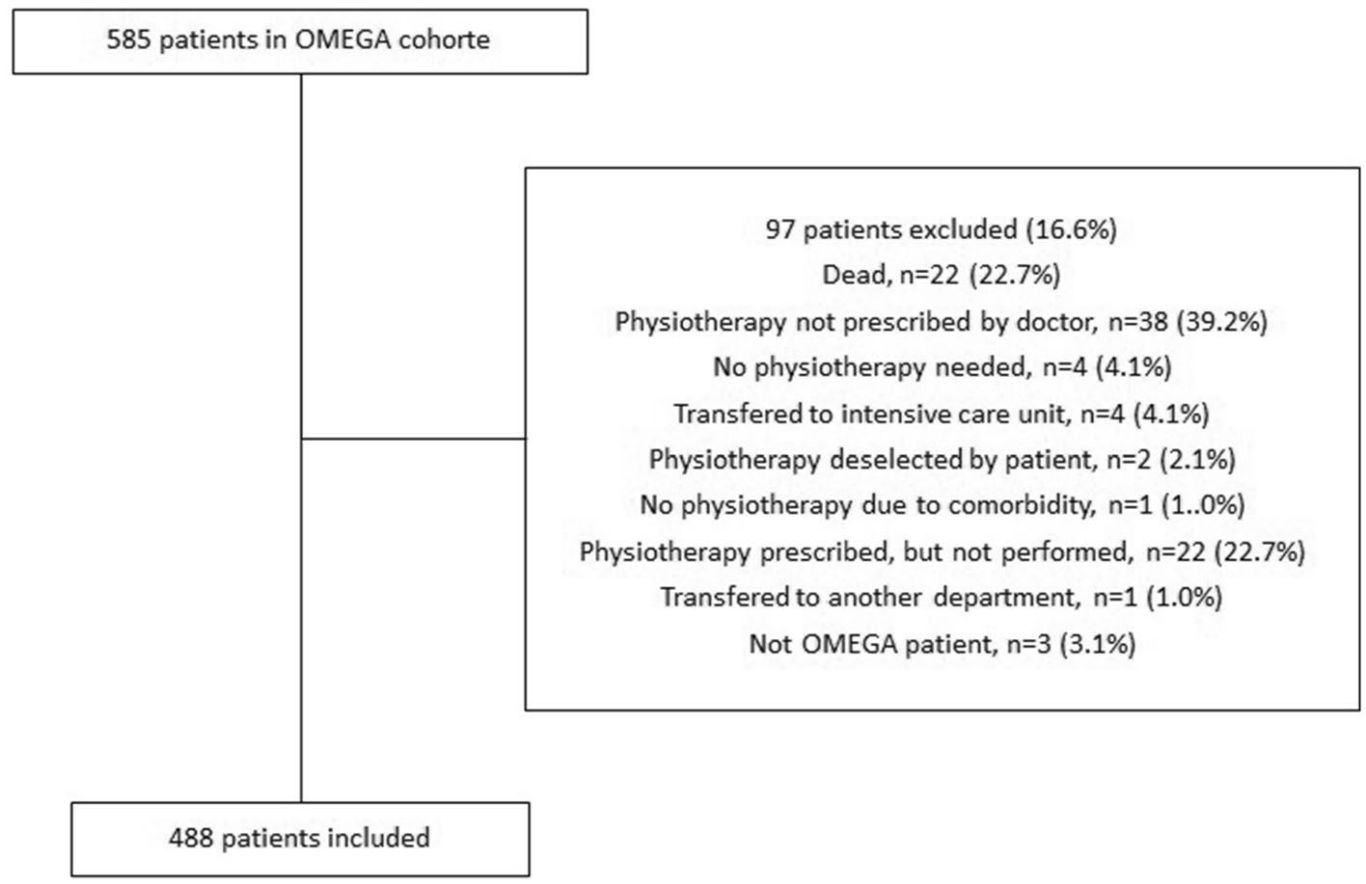
Flowchart of patient inclusion

Five hundred eighty-five patients were eligible for inclusion in this study. A total of 97 patients were excluded due to the lack of postoperative physiotherapeutic assessment during admission. The reasons for no postoperative physiotherapeutic assessment are represented in Fig. 1. The total number of patients included was 488, with a median age of 69% and 50.6% male. Patient characteristics are presented in Table 1. Most patients (74.2%) had physiotherapeutic assessment within 0–2 days postoperatively with a median time to assessment of 2.0 days (IQR 1.0–3.0). Physiotherapy lasted a median of 5 days (IQR 5–12). The mean time to mobilization was 1.8 days [standard deviation (SD) 1.5], with 265 patients (55.9%) being mobilized on day 0 or 1.

**Table 1:** Demographics and Clinical Characteristics

Patient characteristics	*n* = 488
Sex, male	247 (50.6)
Age, median (interquartile range), years	69 (58–78)
Age categories	
< 60	135 (27.7)
60–69	110 (22.5)
70–79	156 (32.0)
≥ 80	87 (17.8)
Comorbidities	
Body mass index (kg/m2), median (interquartile range)	25.0 (22.0–29.2)
Respiratory comorbidity	74 (15.2)
Smoking	110 (22.5)
Hypertension	245 (50.2)
Cardiac failure	20 (4.1)
Cardiac ischemia (present or history with)	30 (6.1)
Diabetes	49 (10.0)
Cancer	39 (8.0)
ASA physical status	
1	63 (12.9)
2	217 (44.5)
3	189 (38.7)
4	18 (3.7)
5	1 (0.2)
WHO performance score	
0	240 (49.2)
1	150 (30.7)
2	60 (14.1)
3	24 (4.9)
4	5 (1.0)
Procedures	
Upper GI	40 (8.2)
Small bowel with resection	61 (12.5)
Colon with resection	69 (14.1)
Small bowel and colon with resection	19 (3.9)
Laparotomy without bowel resection	287 (58.8)
Other	12 (2.5)
Open/Laparoscopic procedure	
Open	349 (71.5)
Laparoscopic	56 (11.5)
Laparoscopic converted to open	83 (17.0)

Values are number of patients (%) unless stated otherwise. *COPD* chronic obstructive pulmonary disease. *ASA* American Society of Anaesthesiologists. WHO World Health Organization. Upper GI includes all procedure on the ventricle and the duodenum. Other procedures being appendectomy, splenectomy, salpingectomy, ureteral reimplantation, ruptured spleen, urinary bladder suture, orchiectomy

### Feasibility

A total of 332 (68%) patients underwent DEMMI scoring at the initiation of physiotherapy. A total of 82 patients (16.8%) had two DEMMI scorings, and of these, 75 (91.5%) patients had a higher score at discharge. Hand grip strength was measured in 298 patients (61.1%) at the initiation of physiotherapy. A total of 77 (15.8%) patients furthermore underwent hand grip strength measurement at discharge. Out of these, 22 (28.6%) patients had a worsened score, 28 (36.4%) patients had a heightened score, and 27 (35.0%) patients had no change. The 30-s chair-stand test was performed by 103 patients (21.1%) at the initiation of physiotherapy. A total of 27.3% of patients who underwent laparotomy performed the test and a total of 30.4% of patients who underwent laparoscopic surgery performed the test (*p* = 0.632) A total of 36 patients (7.4%) had an additional scoring at discharge, and of these, 21 patients had a higher number of stands, four patients had worsened, and 11 patients had the same result.

### DEMMI score

The median initiation DEMMI score was 41 (IQR 24.8–53.0). A total of 161 (48.5%) patients had a high score (> 40), and 171 (51.5%) patients had a low score (≤ 40). Patient characteristics according to the DEMMI score are presented in Supplementary Table 2. A comparison of clinical outcomes in patients with high vs. low DEMMI score is presented in Table 2. The multivariate analysis found that the DEMMI score and the ASA classification, were independent risk factors for development of significant postoperative complications CD ≥ 3 (Table 3).

**Table 2:** Comparison of outcomes in patients with high vs. low physical performance scores

	DEMMI > 40 *n* = 161	DEMMI ≤ 40 *n* = 171	*p* value
Postoperative complication CD grade ≥ 3	44 (25.7)	76 (47.2)	** < 0.001**
Length of stay, days (IQR)	5 (4–8)	8 (5–14)	** < 0.001**
Discharged with a rehabilitation plan	33 (20.8)	65 (47.4)	** < 0.001**
Discharged to own home	142 (88.1)	106 (62.0)	** < 0.001**
30-day mortality	4 (2.3)	22 (13.7)	** < 0.001**
	High handgrip strength *n* = 170	Low handgrip strength *n* = 129	
Postoperative complication CD grade ≥ 3	43 (25.3)	55 (42.6)	**0.002**
Length of stay, days (IQR)	5 (4–9)	8 (6–14)	** < 0.001**
Discharged with a rehabilitation plan	26 (16.6)	64 (56.1)	** < 0.001**
Discharged to own home	132 (84.1)	75 (65.8)	** < 0.001**
30-day mortality	3 (1.8)	20 (15.5)	** < 0.001**

Bold values indicate statistical significance

Values are number of patients (%) unless stated otherwise. *CD* Clavien–Dindo. *IQR* interquartile range

**Table 3:** Multivariate analysis including DEMMI score of independent risk factors for postoperative complications CD ≥ 3

	OR	95% CI	*p* value
Sex, male	1.020	0.632; 1.647	0.934
Age			
< 60	Reference		
60–69	1.087	0.537; 2.199	0.817
70–79	1.019	0.529; 1.963	0.956
≥ 80	1.252	0.580; 2.701	0.567
ASA physical status			
1–2	Reference		
> = 3	2.291	1.351; 3.886	**0.002**
WHO performance score			
0–1	Reference		
> = 2	0.589	0.306; 1.134	0.113
DEMMI score			
> 40	Reference		
≤ 40	2.288	1.351; 3.875	**0.002**
Open/Laparoscopic procedure			
Open	Reference		
Laparoscopic	0.510	0.227; 1.143	0.102

Bold values indicate statistical significance

Values are number of patients (%) unless stated otherwise. *COPD* Chronic obstructive pulmonary disease. *ASA* American Society of Anaesthesiologists. Upper GI includes all procedure on the ventricle and the duodenum. Other procedures being appendectomy, splenectomy, salpingectomy, ureteral reimplantation, ruptured spleen, urinary bladder suture, orchiectomy

### Hand grip strength

The median initiation hand grip strength for the entire cohort was 25 kg (IQR 19–36). A total of 170 patients (56.7%) had a high sex-specific hand grip strength, and 129 patients (43.3%) had a low sex-specific hand grip strength. Patient characteristics according to hand grip strength are presented in Supplementary Table 3. A comparison of clinical outcomes in patients with high vs. low hand grip strength is presented in Table 2. The multivariate analysis found low hand grip strength and ASA physical status to be independent risk factors for postoperative complications CD ≥ 3 (Table 4).

**Table 4:** Multivariate analysis including hand grip strength of independent risk factors for postoperative complications CD ≥ 3

	OR	95% CI	*p* value
Sex, male	0.928	0.554; 1.553	0.775
Age			
< 60	Reference		
60–69	0.844	0.394; 1.808	0.663
70–79	0.742	0.362; 1.520	0.415
≥ 80	0.881	0.347; 1.892	0.627
ASA physical status			
1–2	Reference		
> = 3	2.059	1.1181; 3.591	**0.011**
WHO performance score			
0–1	Reference		
> = 2	0.954	0.477; 1.906	0.893
Hand grip strength			
High	Reference		
Low	2.007	1.121; 3.594	**0.019**
Open/Laparoscopic procedure			
Open	Reference		
Laparoscopic	0.54	0.222; 1.317	0.176

Bold values indicate statistical significance

Values are number of patients (%) unless stated otherwise. *COPD* chronic obstructive pulmonary disease. *ASA* American Society of Anaesthesiologists. Upper GI includes all procedure on the ventricle and the duodenum. Other procedures being appendectomy, splenectomy, salpingectomy, ureteral reimplantation, ruptured spleen, urinary bladder suture, orchiectomy

### 30-s chair-stand test

The median of the 30-s chair-stand test was 0 stands (IQR 0–5). A total of 19 patients (18.5%) scored eight or above stands, and 84 patients (81.5%) scored less than eight. The patients with a low score on the 30-s chair-stand test were more likely to suffer a postoperative complication CD grade ≥ 3 than patients with a high score on the 30-s chair-stand test (27.7% (*n* = 32) vs. 6.3% (*n* = 2), respectively (*p* = 0.029)).

## Discussion

This prospective cohort study on patients undergoing major emergency abdominal surgery found that in a standardized setting, physiotherapeutic evaluation with physical performance tests with DEMMI and hand grip strength in the immediate postoperative period were feasible with 2/3 of patients undergoing DEMMI evaluation and hand grip strength. The 30-s chair-stand test was less viable in this population; only 1/5 of the patients could complete the 30-s chair-stand test during the postoperative period. Low DEMMI score (< 40) and low hand grip strength (< 20 kg for women, < 30 kg for men) were alongside ASA classification independent risk factors for the development of postoperative severe complications CD grade ≥ 3. Low DEMMI and low hand grip strength furthermore showed a tendency towards a longer length of stay, a higher risk of needing postoperative rehabilitation after discharge, and a tenfold higher risk of 30-day mortality.

Only sparse literature on patients undergoing major emergency surgery with postoperative performance test evaluation can be found. A prior study focusing on postoperative physical performance presenting physiotherapeutic data for patients undergoing major emergency abdominal surgery investigated a similar cohort of 50 patients who underwent daily physiotherapy (10–30 min) on postoperative days 1–7. In accordance with our study, 2/3 of their patients were able to perform a physical performance test (Cumulated Ambulation Score) in the postoperative period [5]. The 30-s chair-stand test is physically more demanding, and this test was not feasible in patients undergoing major emergency surgery. These results emphasize the physical impairment these patients suffer from in the immediate postoperative period and the challenges of adhering to standardized postoperative physiotherapy. Our results are in accordance with the previous study by Jønsson et al., where up to 35% of the patients were non-independently mobile and had low levels of 24-h physical activity one week after surgery despite high pre-operative functional levels and generally being healthy [5].

We found that a DEMMI score < 40 is an independent risk factor for developing severe postoperative complications. To our knowledge, no prior studies have investigated the association between DEMMI score and postoperative complications in emergency surgery. In accordance with our results, a previous study on the length of stay for geriatric patients admitted to the emergency department found that DEMMI < 40 was associated with a longer hospital length of stay [9]. A low DEMMI score indicated impaired mobilization, and a previous study in patients undergoing abdominal surgery found delayed mobilization associated with postoperative pulmonary complications [19]. Low hand grip has to our knowledge, never been investigated in patients undergoing emergency surgery; however, low preoperative hand grip strength has previously been associated with impaired postoperative recovery and quality of life, morbidity, and mortality in elective orthopaedic and general surgical procedures [20–24]. Hand grip strength is a muscle strength marker and part of the sarcopenia syndrome [8]. Surgery-induced inflammation is increased in patients with reduced muscle endurance[25] and sarcopenia has previously been associated with morbidity and mortality following general emergency surgery [26–30].

In our study, 31.1% of the patients were discharged with a rehabilitation plan, which means their physical recovery after admission has given medical justification to continue rehabilitation in the patient's municipality facilities. Patients discharged with a rehabilitation plan have thus experienced a significant loss of function during admission. This suggests that the consequences and need for rehabilitation after major emergency abdominal surgery reaches beyond the immediate postoperative period and hospitalization. A study with a population of patients recovering from a hip fracture found that functional physical recovery might be complete within the first six months; however, emotional recovery may extend for up to 9 months after surgery [31]. Previous studies have found that patients of high age have a good quality of life and have returned to their preadmission residential status six months after emergency abdominal surgery [32, 33]. This could indicate that patients undergoing major emergency abdominal surgery could benefit from a more extensive follow-up plan after discharge and up to six months postoperative.

There are several limitations to this study. The data came from a single centre and might not be completely generalizable to other study populations. The physical performance tests (DEMMI-score, hand grip strength, 30-s chair-stand test) were not performed by all eligible patients; why results from these tests are not representable of all patients undergoing major emergency surgery, but instead they represent results for patients where the surgeon prescribed physiotherapy indicating a clinical need for physiotherapy and the patients able to perform physical performance test after major emergency abdominal surgery. This, however, further emphasizes the physiological loss of function in this patient group. The study is strengthened by the prospective data collection and by including a relatively high number of patients.

The physical impairment and the difficulties of postoperative mobilization after major emergency surgery were illustrated in this study, and future research should focus on these challenges. These results can both be used in the clinical setting or in future research like prediction models predicting outcomes for patients undergoing emergency surgery. Both DEMMI score and hand grip strength are tests that can identify vulnerable patients. Using physical performance tests to identify at-risk patients, postoperative interventions can be aimed at patients in need. Delayed postoperative mobilization has been associated with postoperative pulmonary complications[19], and early mobilization is an integrated part of ERAS protocols for patients undergoing elective surgery and these principles can be applied in emergency surgical patients postoperatively [34]. Furthermore, goal-directed mobilization in patients in the intensive care unit (ICU) has been found to reduce ICU stay and improve functional mobility at discharge [35]. No standardized postoperative bundle of care exists for patients undergoing major emergency abdominal surgery, and an increased focus on mobilization and enhanced recovery in this patient population is needed.

In conclusion, DEMMI score and hand grip strength are feasible performance tests to use postoperatively in evaluating patients undergoing major emergency abdominal surgery. Low DEMMI and low hand grip strength were associated with the development of severe postoperative complications.

### Supplementary Information

Below is the link to the electronic supplementary material.

Supplementary file1 (PDF 189 KB)
