# Supplementary material for: Association between reduced physical performance measures and short-term consequences after major emergency abdominal surgery: a prospective cohort study
Source: Eur J Trauma Emerg Surg. 2024 Jan 5;50(3):821–8. doi: 10.1007/s00068-023-02408-4 (PMC11249428; doi:10.1007/s00068-023-02408-4)
Supplement: Supplementary file 1 — Supplementary file1 (PDF 189 KB) [file 68_2023_2408_MOESM1_ESM.pdf]

Title

Association between reduced physical performance measures and short-term consequences after major emergency abdominal surgery: a prospective cohort study

Journal

European Journal of Trauma and Emergency Surgery

Authors

Dunja Kokotovic, Aide Schucany, Liv Soylu, Andreas Q Fenger, Iben Puggard, Sarah Ekeloef, Ismail Gögenur, Jakob Burcharth

Corresponding author

Dunja Kokotovic

Borgmester Ib Juuls Vej 1, 2730 Herlev, Denmark

Telephone: +45 28 77 63 64

E-mail: Dunja.kokotovic@hotmail.com

Supplementary Table 1. Definition of emergency abdominal surgery

Inclusion criteria
• Open, laparoscopic, or laparoscopically-assisted procedures<br>• Procedures involving the stomach, small or large bowel, or rectum for conditions such as perforation, ischemia, abdominal abscess, bleeding or obstruction<br>• Washout/evacuation of intra-peritoneal abscess<br>• Washout/evacuation of intra-peritoneal hematoma<br>• Bowel resection/repair due to incarcerated umbilical, inguinal and femoral hernias (but not hernia repair without bowel resection/repair)<br>• Bowel resection/repair due to obstructing/incarcerated incisional hernias provided the presentation and findings were acute<br>• Laparotomy/laparoscopy with inoperable pathology (e.g. peritoneal/hepatic metastases)<br>• Laparoscopic/Open adhesiolysis<br>• Return to theatre for the repair of fascial dehiscence<br>• Any reoperation/return to theatre meeting the criteria above is included
Exclusion criteria
• Elective laparoscopy or laparotomy<br>• Diagnostic laparoscopy where no subsequent procedure is performed<br>• Appendectomy +/- drainage of localized collection unless the procedure is incidental to a non-elective procedure on the GI tract<br>• Cholecystectomy +/- drainage of localized collection unless the procedure is incidental to a non-elective procedure on the GI tract<br>• All surgery involving the appendix or gallbladder, including any surgery relating to complications such as abscess or bile leak, is excluded<br>• Non-elective hernia repair without bowel resection.<br>• Large-bowel stent procedures<br>• Superficial wound dehiscence above the fascia<br>• Ruptured ectopic pregnancy, or pelvic abscesses due to pelvic inflammatory disease<br>• Laparotomy/laparoscopy for pathology caused by blunt or penetrating trauma<br>• Laparotomy/laparoscopy for esophageal pathology<br>• Laparotomy/laparoscopy for pathology of the spleen, renal tract, kidneys, liver, gall bladder, and biliary tree, pancreas, or urinary tract

Supplementary Table 2. Univariate comparison of patients with high (> 40) vs low (≤ 40) DEMMI score

Patient characteristics	DEMMI > 40<br>n = 161	DEMMI ≤ 40<br>n = 171	*p*-value
Sex, male	92 (53.8)	68 (42.2)	0.035
Age			
< 60	61 (35.7)	29 (18.0)	< 0.001
60 - 69	42 (24.6)	31 (19.3)	
70 - 79	53 (31.0)	55 (34.1)	
≥ 80	15 (8.8)	46 (28.6)	
Body mass index (kg/m2) > 25	91 (54.2)	75 (47.5)	0.227
Respiratory comorbidity	16 (9.4)	31 (19.3)	0.010
Smoking	29 (19.5)	38 (26.8)	0.139
Hypertension	70 (40.9)	93 (57.8)	0.002
Cardiac failure	5 (2.9)	10 (6.2)	0.150
Cardiac ischemia (present or history with)	7 (4.1)	14 (8.7)	0.085
Diabetes	10 (5.8)	19 (11.8)	0.055
Cancer	11 (6.4)	15 (9.3)	0.328
ASA physical status			< 0.001
1-2	121 (70.8)	70 (43.5)	
>= 3	50 (29.2)	91 (56.5)	
WHO performance score			< 0.001
0-1	162 (94.7)	103 (64.0)	
>= 2	9 (5.3)	58 (36.0)	
Procedures			0.578
Upper GI	13 (7.6)	14 (8.7)	
Small bowel with resection	22 (12.9)	17 (10.6)	
Colon with resection	18 (10.5)	28 (17.4)	
Small bowel and colon with resection	8 (4.7)	7 (4.3)	
Laparotomy without bowel resection	107 (62.6)	93 (57.8)	
Other	3 (1.8)	2 (1.2)	
Open/Laparoscopic procedure			0.015
Open	142 (83.0)	148 (91.9)	
Laparoscopic	29 (17.0)	13 (8.1)	

Values are number of patients (%) unless stated otherwise. COPD = Chronic obstructive pulmonary disease. ASA = American Society of Anaesthesiologists. WHO = World Health Organization. Upper GI includes all procedure on the ventricle and the duodenum. Other procedures being appendectomy, splenectomy, salpingectomy, ureteral reimplantation, ruptured spleen, urinary bladder suture, orchiectomy.

Supplementary Table 3. Univariate comparison of patients with high vs low hand grip strength

Patient characteristics	High grip strength<br>n = 170	Low grip strength<br>n = 129	*p*-value
Sex, male	94 (55.3)	48 (37.2)	0.002
Age			
< 60	75 (44.1)	14 (10.9)	< 0.001
60 - 69	41 (24.1)	19 (14.7)	
70 - 79	42 (24.7)	55 (42.6)	
≥ 80	12 (7.1)	41 (31.8)	
Body mass index (kg/m2) > 25	100 (60.2)	44 (34.6)	< 0.001
Respiratory comorbidity	21 (12.4)	22 (17.1)	0.251
Smoking	36 (24.5)	21 (18.9)	0.286
Hypertension	64 (37.6)	77 (59.7)	< 0.001
Cardiac failure	5 (2.9)	8 (6.2)	0.171
Cardiac ischemia (present or history with)	6 (3.5)	12 (9.3)	0.038
Diabetes	13 (7.6)	9 (7.0)	0.826
Cancer	9 (5.3)	12 (9.3)	0.179
ASA physical status			< 0.001
1-2	156 (91.8)	90 (69.8)	
>= 3	14 (8.2)	39 (30.2)	
WHO performance score			< 0.001
0-1	162 (94.7)	103 (64.0)	
>= 2	9 (5.3)	58 (36.0)	
Procedures			0.377
Upper GI	13 (7.6)	11 (8.5)	
Small bowel with resection	21 (12.4)	16 (12.4)	
Colon with resection	14 (8.2)	21 (16.3)	
Small bowel and colon with resection	8 (4.7)	7 (5.4)	
Laparotomy without bowel resection	110 (64.7)	71 (55.0)	
Other	4 (2.4)	3 (2.3)	
Open/Laparoscopic procedure			0.027
Open	144 (84.7)	120 (93.0)	
Laparoscopic	26 (15.3)	9 (7.0)	

Values are number of patients (%) unless stated otherwise. COPD = Chronic obstructive pulmonary disease. ASA = American Society of Anaesthesiologists. WHO = World Health Organization. Upper GI includes all procedure on the ventricle and the duodenum. Other procedures being appendectomy, splenectomy, salpingectomy, ureteral reimplantation, ruptured spleen, urinary bladder suture, orchiectomy.
